# Predicting lower extremity deep venous thrombosis in patients with aneurysmal subarachnoid hemorrhage: a machine learning study

**DOI:** 10.3389/fneur.2025.1659212

**Published:** 2025-11-24

**Authors:** Fengfeng Jiang, Chenxing Ye, Danfeng Yu, Feng Chen, Wei Xu, Pingyou He, Chengwei Zhang, Minfeng Tong, Xiang Bao

**Affiliations:** 1 Department of Neurosurgery, Affiliated Jinhua Hospital, Zhejiang University School of Medicine, Jinhua, China; 2 Department of Traumatology, Affiliated Hangzhou First People's Hospital, Westlake University School of Medicine, Hangzhou, China

**Keywords:** aneurysmal subarachnoid hemorrhage (aSAH), lower extremity deep venous thrombosis (LEDVT), machine learning (ML), prediction, XGBoost (extreme gradient boosting)

## Abstract

**Background:**

Lower extremity deep venous thrombosis (LEDVT) is a frequent and serious complication after aneurysmal subarachnoid hemorrhage (aSAH). Existing risk scores poorly discriminate LEDVT risk in this population.

**Objective:**

To develop and externally validate machine learning (ML) models for early prediction of LEDVT in aSAH patients treated with endovascular therapy.

**Methods:**

We performed a retrospective multicenter study including an internal cohort (*n* = 593) for model development and internal validation and an external cohort (*n* = 142) for external validation. Thirty-seven clinical and laboratory variables were considered. Variable selection used LASSO followed by multivariable logistic regression. Seven ML algorithms (XGBoost, LightGBM, random forest, logistic regression, SVM, KNN, MLP) were trained with 5 × 5-fold cross-validation; AUC was the primary metric. Model interpretability used SHAP. An online risk calculator was implemented.

**Results:**

Six predictors were selected (age, albumin, D-dimer, GCS, AISI, and MCA aneurysm). XGBoost achieved the best discrimination (internal AUC 0.88; external AUC 0.80). Decision curve analysis showed clinical net benefit across relevant thresholds. SHAP analysis highlighted D-dimer, albumin, and GCS as key contributors. A web-based calculator was deployed to facilitate clinical use.

**Conclusions:**

An XGBoost-based model incorporating six routinely available variables accurately predicts LEDVT risk after aSAH and generalized to an external cohort. The web tool may help target preventive strategies for high-risk patients.

## 1 Introduction

Aneurysmal subarachnoid hemorrhage (aSAH) caused by intracranial aneurysm rupture is a severe neurologic emergency with devastating effects and unfavorable outcomes. It is demonstrated that the case fatality of aSAH can be as high as 30%−50% and at least 20% of those who do survive are unable to regain functional independence (1–4). Since the International Subarachnoid Aneurysm Trial (ISAT), endovascular treatment (EVT) has become the preferred first-line therapy for intracranial aneurysms, as it is less invasive and improves postoperative quality of life (5–7). Despite advances in aneurysm treatment, complications such as lower extremity deep vein thrombosis (LEDVT) remain difficult to prevent. LEDVT can cause limb swelling, pain, and varicose veins, and may even lead to pulmonary embolism (PE), posing a serious risk for aSAH patients.

Therefore, it is urgently necessary to identify and predict aSAH patients who are at the highest risk of developing lower extremity DVT as early as possible. The pathogenesis of LEDVT is well understood, and scores are now available to predict its occurrence; for example, the Caprini score can assess the risk of DVT based on patient age, BMI, and medical history (8). However, according to the criteria of this scoring system, all patients with aSAH are classified as extremely high-risk DVT patients. This limitation makes it difficult to distinguish which patients are truly at higher risk and therefore in greater need of targeted preventive strategies. On the other hand, the Caprini score are not very suitable for aSAH patients. For instance, since aSAH patients are often bedridden for prolonged periods, accurately measuring their BMI is challenging, which compromises the precision of their Caprini risk assessment. Moreover, critical indicators such as the GCS, Hunt-Hess, and Fisher grades—used to evaluate aSAH severity—are not incorporated into the Caprini scoring system. Therefore, In 2023, our team pioneered the development of a prognostic nomogram to predict LEDVT risk in aSAH patients undergoing endovascular treatment, demonstrating promising preliminary results. In addition, recent studies have identified several hematologic and inflammatory markers, such as neutrophil-to-lymphocyte ratio (NLR) (9), the inflammatory burden index (IBI) (10), systemic immune inflammation index (SII) (11), systemic inflammatory response index (SIRI); aggregate index of systemic inflammation (AISI), and hemoglobin concentration (12), as significant predictors of DVT. These advancements highlight the need to further refine predictive models by incorporating additional inflammatory and clinical markers.

In addition, recent research has underscored the potential of machine learning (ML) algorithms in predicting stroke-related complications (13, 14). While conventional logistic regression (LR) offers interpretable models for clinical prediction, it often struggles with complex, non-linear, and multivariate relationships due to issues such as multicollinearity and low robustness—requiring extensive data transformations (15). As ML techniques gain popularity and research into LEDVT prediction deepens, several ML-based models have been successfully developed for this purpose. LEDVT prediction models based on various ML techniques have been successfully constructed and developed (16). Previously, Li et al. (15) utilized machine learning models for predicting postoperative pneumonia, and they achieved better prediction results than traditional modeling. Thus, in this study, we conducted six ML prediction model, which contains Linear models [logistic regression (LR), support vector machine (SVM)] and Non-linear models (XGBoost, k-nearest neighbor (KNN), random forest (RF), light gradient boosting machine (LightGBM) and multilayer perceptron (MLP) for the prediction of LEDVT within 30 days in aSAH patients.

## 2 Materials and methods

### 2.1 Study population

The retrospective study was conducted according to STROBE guidelines and all procedures adhered to relevant ethical and regulatory standards (17). The aSAH data were derived from two hospital databases: Internal cohort for training and testing model, and external cohort for validating model. Internal data were derived from the First Affiliated Hospital of Wenzhou Medical University from January 1, 2020 to December 31, 2022. The external cohort was extracted from the Aneurysm Data Center of Hangzhou First People's Hospital from January 1, 2022 to December 31, 2022. Study samples and treatment data were retrieved from the respective surgical department databases. All included aSAH patients had symptom onset within 72 h and were diagnosed using computed tomography (CT), computed tomography angiography (CTA), or digital subtraction angiography (DSA) (8, 18, 19).

Patients in the internal cohort were excluded if they met any of the following criteria: (1) Age < 18 (*N* = 4); (2) Presence of intracranial vascular malformations or moyamoya disease (*N* = 29); (3) Patients with preoperative unexpected events that could affect the outcome: preoperative cardiac arrest (*N* = 1), severe head trauma (*N* = 2); (4) Intraoperative or postoperative rebleeding (*N* = 14); (5) Missing critical information (*N* = 4); (6) Positive SARS-CoV-2 PCR result (*N* = 0). Patients with any missing data for the analyzed variables were excluded to ensure a complete-case analysis. Finally, the internal cohort (*n* = 593) was randomly split into a training set (*n* = 474, 80%) and an internal testing/validation set (*n* = 119, 20%).

**Figure 1:**
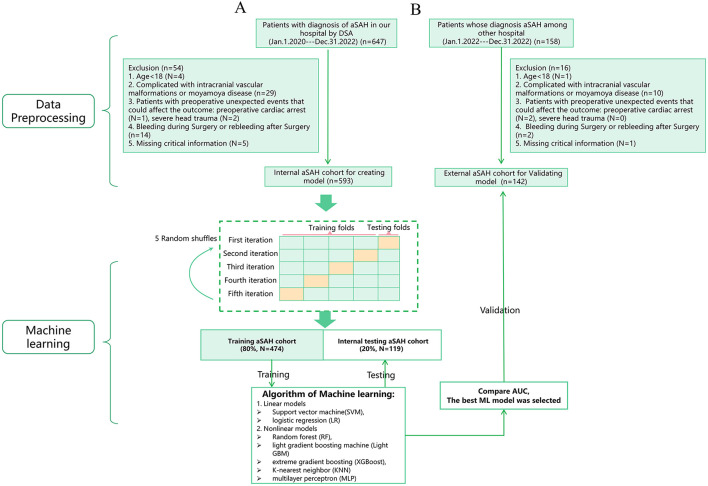
Flowchart of the internal cohort **(A)** and external cohort **(B)**.

Patients in the external cohort were excluded based on the following criteria: (1) Age < 18 (*N* = 1); (2) complicated with intracranial vascular malformations or moyamoya disease (*N* = 10); (3) Patients with preoperative unexpected events that could affect the outcome: preoperative cardiac arrest (*N* = 2), severe head trauma (*N* = 0); (4) Bleeding during Surgery or rebleeding after Surgery (*N* = 2); (5) Missing critical information (*N* = 1); (6) The SARS-CoV-2 PCR results were positive (*N* = 0). Finally, a total of 142 cases were enrolled for validating cohort. Figure 1 shows the flow diagram of the data processing in detail.

### 2.2 Ethical approval

The data used in this study were approved by the Biomedical Research Ethics Committee of Wenzhou Medical University (BR/NGGI-2412031) and Medical Ethics Review Board of Hangzhou First People's Hospital (ME/lXGS6-105). Informed consent was waived due to the retrospective design of the study and the absence of personal identifiers, in accordance with the principles of the Declaration of Helsinki (20).

### 2.3 Variable definition and selection

The diagnostic criteria for LEDVT were based on Doppler ultrasound (DUS) findings of intravascular shadows in the deep veins of the lower limbs. Examinations included B-mode imaging and color Doppler flow imaging, with or without probe compression, performed within 2 weeks post-surgery (21, 22). Given the high incidence of lower extremity DVT, each patient was examined weekly by DUS following hospital admission. Collected variables included: (1) patients demographics (age, gender, history of smoking, history of alcohol use); (2) GCS, Hunt-Hess grades, modified Fisher (mFS) grade and WFNS grade on admission; (3) past medical history (hypertension, diabetes mellitus, coronary heart disease (CHD), infection; (4) aneurysm location [anterior communicating artery (ACoA), internal carotid artery (ICA), middle cerebral artery (MCA), posterior communicating artery (PCoA), vertebrobasilar artery (VBA)]; (5) laboratory results were obtained within 24 h after admission in the context of a first examination (albumin, hemoglobin, neutrophils, monocytes, lymphocytes, uric acid, total cholesterol, triglycerides, neutrophils, monocytes, mean corpuscular volumec-reactive (MCV), C-reactive protein (CRP), and the composite index calculated by these laboratory results: Inflammatory Burden Index (IBI) = CRP × neutrophils/lymphocytes (23), SII = total number of neutrophils × total number of platelets/total number of lymphocytes, SIRI = total number of monocytes × total number of platelets)/total number of lymphocytes, AISI = total number of neutrophils × total number of monocytes × total number of platelets)/total number of lymphocytes (24).

The machine learning model developed to predict LEDVT risk in aSAH patients relies on statistically significant factors identified through multivariate logistic regression analysis (*p* < 0.05) (25).

### 2.4 ML model construction

The patients were randomly divided into a training cohort (*n* = 474) and a validation cohort (*n* = 119) in an 80:20 ratio. The training cohort was used to develop both linear models [logistic regression (LR), support vector machine (SVM)] and non-linear models [XGBoost, k-nearest neighbors (KNN), random forest (RF), light gradient boosting machine (LightGBM), and multilayer perceptron (MLP)] (26–28). XGBoost model was constructed using the xgboost package (https://xgboost.readthedocs.io/en/latest/python/index.html). The remaining five models were established via Scikit-learn package (https://github.com/scikit-learn/scikit-learn). The best hyperparameter combination for each model (provided in Supplementary Table S1) was determined using a grid search strategy with 5-fold cross-validation on the training cohort, employing the area under the curve (AUC) as the primary optimization metric. To develop an unbiased assessment of model performance, we performed 5 random shuffles of 5-fold cross-validation, as shown in Figure 1. Each iteration used a different stratified fold for model evaluation, and the remaining folds were used for model training (29). Subsequently, we recorded area under the curve (AUC) accuracy, sensitivity, specificity, F1 score, and Kappa to compare each ML model. AUC was used as the primary metric for model selection. The best-performing model was then validated using the external dataset. Then it is tested on external data. Data processing and the ML process are summarized in Figure 1.

To investigate the impact of class imbalance (10.8% LEDVT-positive cases) on model performance, a sensitivity analysis was conducted using the Synthetic Minority Over-sampling Technique (SMOTE) (30). This method was applied to the training data to generate synthetic samples for the minority class, creating a balanced 1:1 class distribution for model training.

After the model was established, the SHapley Additive exPlanations (SHAP) package in Python was used to explain the model by analyzing two cases. The SHAP package interpreted the output of the machine learning model using a game-theoretic approach (31). SHAP values quantify the association of a variable with the outcome of a single patient, and the mean absolute SHAP value across all patients is reported as the SHAP value of variable (32). The overall importance of each feature was determined by ranking them according to their mean absolute SHAP value, which represents the average magnitude of each feature's impact on the model output across all samples. This approach provides a consistent and theoretically grounded method for feature importance assessment.

### 2.5 Statistical analysis

Categorical variables were analyzed using the Chi-square test. For continuous variables, if the distribution is normal and the variance is homogeneous, the *t*-test would be used. The Welch's *t*-test would be used when the normal distribution was met but the variance was not homogeneous. For non-normally distributed variables, the Mann–Whitney *U* test was used. Logistic regression analysis was used to estimate univariate and multivariate odds ratios and 95% confidence intervals. A *p*-value < 0.05 was considered statistically significant. The concordance index (C-index) was used to assess the discriminative ability of the model. The optimal probability threshold for classifying LEDVT risk was determined by maximizing Youden's index (*J* = sensitivity + specificity – 1) based on the ROC curve of the training set. Calibration curves were used to assess the agreement between predicted and observed risks. Model calibration was further evaluated using the Hosmer–Lemeshow goodness-of-fit test (33). Clinical utility and net benefits were determined by decision curve analysis (DCA) (34). 1,000 bootstrap resamples were used for external validation, and the relative corrected C-index was calculated to ensure the stability of the nomogram in the external validation cohort. All tests of significance were two-sided, and a *p* value of < 0.05 was considered statistically significant. All statistical analyses were performed using R version 3.6.3 and python version 3.7.

## 3 Results

### 3.1 Demographic characteristics

Table 1 shows the clinical characteristics of the study population. A total of 593 aSAH patients were included in this study. The number of patients with LEDVT was 106 (16%), and men comprised 108 (34%) and 54 (40%) patients in the two groups, respectively. The median ages of the non-LEDVT and LEDVT cohorts were 55 and 64 years, respectively. The LEDVT cohort had a longer length of hospital stay (LOS) compared to the non-LEDVT cohort (19 vs. 14 days, *p* = 0.003).

**Table 1:** Characteristics of the study population.

**Patient characteristics**	**Total (*n* = 593)**	**No LEDVT (*n* = 529)**	**LEDVT (*n* = 64)**	**p value**
Age, median [IQR]	56.00 [48.00,66.00]	55.00 [48.00,65.00]	64.00 [55.00,71.00]	**< 0.001**
Gender(male), ***n*** (%)	206(34.74)	180(34.03)	26(40.63)	0.295
Smoking history, *n* (%)	90(15.18)	81(15.31)	9(14.06)	0.792
LOS (day), median [IQR]	14.00[11.00,19.00]	14.00[11.00,19.00]	19.00[11.00,24.00]	**0.003**
**In-hospital complications**				
Hypertension, *n* (%)	311 (52.45)	275 (51.98)	36 (56.25)	0.519
Heart Disease, *n* (%)	18 (3.04)	16 (3.02)	2 (3.13)	0.965
Diabetes, *n* (%)	42 (7.08)	38 (7.18)	4 (6.25)	0.783
Pulmonary infection, *n* (%)	103 (17.37)	80 (15.12)	23 (35.94)	**< 0.001**
DCI, *n* (%)	37 (6.24)	30 (5.67)	7 (10.94)	0.100
Timing to DCI (day), mean (±SD)	6.1 ± 1.9	6.0 ± 2.0	6.3 ± 2.0	0.768
**Aneurysm location**				
VBA aneurysm, *n* (%)	29 (4.89)	24 (4.54)	5 (7.81)	0.251
MCA aneurysm, *n* (%)	115 (19.39)	89 (16.82)	26 (40.63)	**< 0.001**
ICA aneurysm, *n* (%)	172 (29.01)	157 (29.68)	15 (23.44)	0.299
PCoA aneurysm, *n* (%)	142 (23.95)	128 (24.20)	14 (21.88)	0.955
ACoA aneurysm, *n* (%)	190 (32.04)	163 (30.81)	27 (42.19)	0.065
**Admission clinical grade**				
GCS, median [IQR]	15.00[15.00,15.00]	15.00[15.00,15.00]	13.00[8.00,15.00]	**< 0.001**
Hunt-Hess grades 4–5, *n* (%)	71 (11.97)	45 (8.51)	26 (40.63)	**< 0.001**
WFNS grade 4–5, *n* (%)	104 (17.54)	74 (13.99)	30 (46.88)	**< 0.001**
mFS grade 3–4, *n* (%)	178 (30.02)	146 (27.60)	32 (50.00)	**< 0.001**
**Treatment modality**				
Surgical clipping, *n* (%)	76 (12.82)	59 (11.15)	17 (26.56)	**< 0.001**
Endovascular coiling, *n* (%)	517 (87.18)	470 (88.85)	47 (73.44)	**< 0.001**
Anticoagulation, *n* (%)	293 (49.41)	271 (51.22)	22 (34.38)	**0.002**
**Laboratory values on admission**				
Albumin (g/L), median [IQR]	39.00 [36.50,41.50]	39.20 [37.00,41.50]	36.90 [31.50,40.20]	**< 0.001**
Glucose (mmlo^/^L), median[IQR]	6.40 [5.40,7.60]	6.30 [5.30,7.60]	6.90 [6.00,8.10]	**0.007**
Triglyceride (μmlo^/^L), median [IQR]	1.10 [0.83,1.69]	1.10 [0.82,1.69]	1.10 [0.89,1.50]	0.860
Uric acid (μmlo^/^L), median [IQR]	242.00 [176.00,302.00]	242.00[176.00,301.00]	243.00 [185.00,306.00]	0.917
Total cholesterol (μmlo^/^L), median [IQR]	4.89 [4.30,5.67]	4.95 [4.37,5.67]	4.47 [4.07,5.33]	**0.037**
Neutrophil counts (^*^10^9/^L), median [IQR]	9.20 [6.53,12.10]	9.08 [6.52,11.78]	10.47 [7.43,14.24]	**0.049**
Monocyte count (^*^10^9/^L), median [IQR]	0.49 [0.33,0.73]	0.48 [0.32,0.71]	0.62 [0.39,0.98]	**0.015**
Lymphocyte count (^*^10^9/^L), median [IQR]	1.14 [0.84,1.57]	1.14 [0.85,1.58]	1.12 [0.73,1.26]	0.091
Hemoglobin (g/L), median [IQR]	132 [119,142]	132.00 [121,142]	123.00 [113,140]	**0.026**
MCV,(fl), median [IQR]	89.8 [86.9,92.8]	89.6 [86.8,92.7]	90.9 [88.7,94.0]	**0.038**
Blood platelet count (^*^10^9/^L), median [IQR]	213 [175,258]	217.00 [176,257]	192.00 [148,264]	**0.063**
CRP(mg/L), median [IQR]	5.00 [1.90,10.20]	5.00 [1.65,9.50]	5.80 [3.69,12.20]	**0.003**
D-dimer(μg/ml), median [IQR]	1.23 [0.56,2.69]	1.07 [0.52,2.34]	4.30 [2.48,7.00]	**< 0.001**
IBI, median [IQR]	37.14 [13.88,79.20]	33.52 [12.79,76.81]	56.46 [40.78,127.70]	**< 0.001**
SII, median [IQR]	1,298.57 [435.08, 2501.67]	1,298.57 [443.09, 2481.01]	1,355.92 [258.00, 2691.80]	0.906
SIRI, median [IQR]	3.42 [1.86, 6.93]	3.33 [1.86, 6.50]	6.96 [2.19, 9.37]	**0.002**
NLR, median [IQR]	25.99 [17.79, 39.13]	26.35 [17.79, 39.13]	24.10 [17.94, 35.64]	0.165

LOS, length of stay; ACoA, anterior communicating artery; PCoA, posterior communicating artery; ICA, internal carotid artery; MCA, middle cerebral artery; VBA, vertebrobasilar aneurysm; CRP, C-reactive protein; MCV, mean corpuscular volume; GCS, Glasgow coma scale; WFNS, World federation of neurosurgical societies; Stent-Assisted EVT, stent assisted endovascular treatment; DCI, delayed cerebral ischemia; AISI, aggregate index of systemic inflammation; NLR, neutrophil-to-lymphocyte ratio; IBI, inflammatory burden index; SII, systemic immune inflammation index; LEDVT, lower extremity deep vein thrombosis. Bold values indicate statistically significant differences (*p* < 0.05).

### 3.2 Feature selection

**Figure 2:**
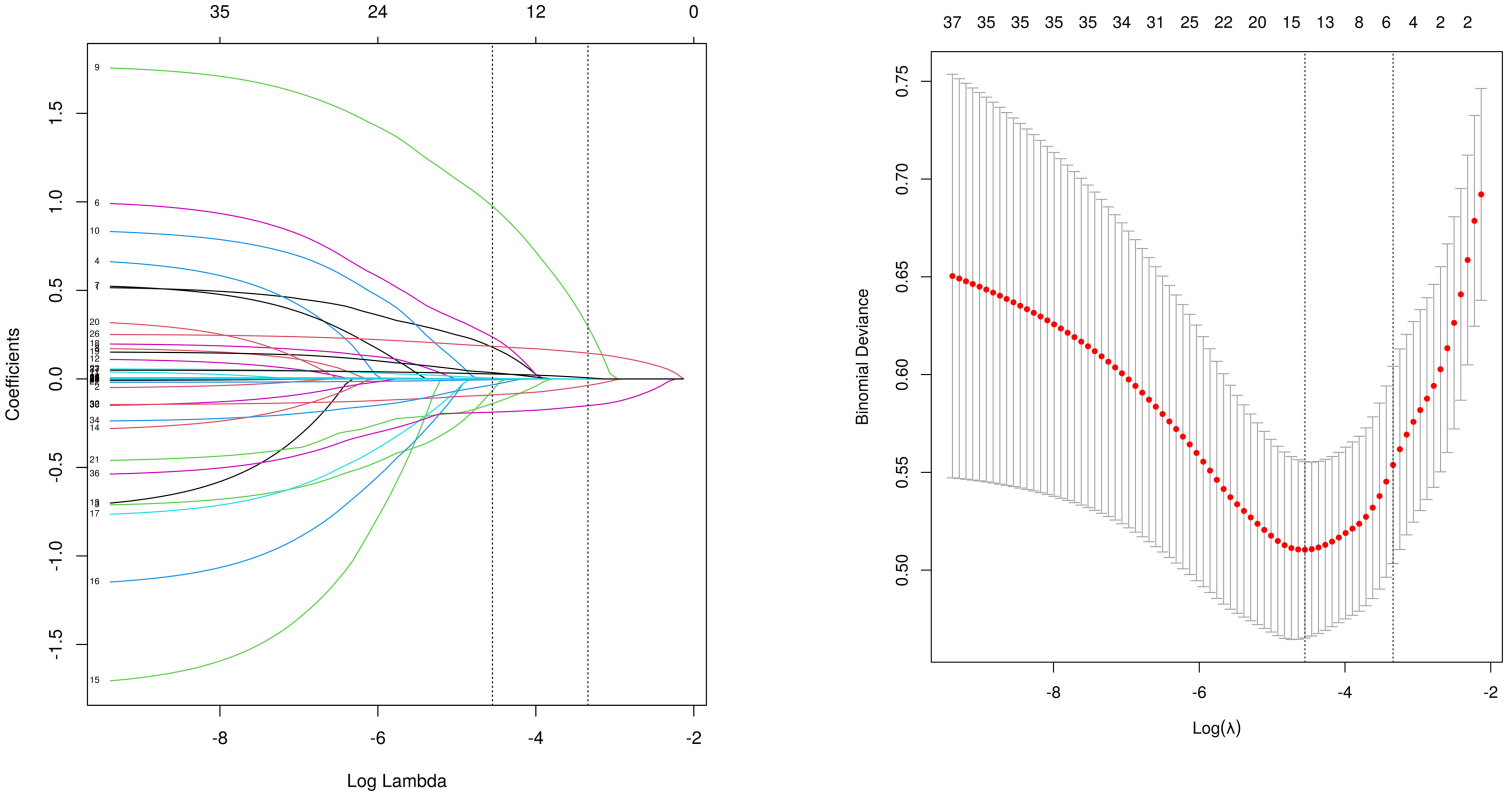
Patient characteristics selection using a LASSO logistic regression model. **(A)** The minimum criteria (lambda.min) and 1 SE of the minimum criteria (lambda. 1se) were used to depict the optimal values with dotted vertical lines. **(B)** LASSO coefficient profile of 37 variables. The coefficient profile is plotted according to the logarithmic sequence. To determine the optimal predictors of the model, five-fold cross-validation with minimum criteria was used, resulting in six features (MCA + D-dimer + AISI + Albumin + GCS + Age) with nonzero coefficients were selected. GCS, Glasgow coma scale; AISI, aggregate index of systemic inflammation; MCA, middle cerebral artery aneurysm.

Of the 37 variables initially collected, 6 were selected using least absolute shrinkage and selection operator (LASSO) regression based on non-zero coefficients (Figure 2). These six variables were subsequently entered into a multivariable logistic regression model, and all were found to be independently associated with LEDVT. These predictors were used to construct the final risk scoring model (Table 2). These variables include age (OR, 1.05; 95% CI, 1.02–1.09; *p* = 0.002), albumin level (OR, 0.89; 95% CI, 0.83–0.96; *p* = 0.001), D-dimer level (OR, 1.28; 95% CI, 1.17–1.42; *p* < 0.001), GCS score (OR, 0.82; 95% CI, 0.74–0.91; *p* < 0.001), Middle cerebral artery (MCA) aneurysm (OR, 3.16; 95% CI, 1.38–7.18; *p* = 0.006), AISI (OR, 1.02; 95% CI, 1.01–1.04; *p* = 0.017). To determine the individual predictive values, we used ROC curve analysis in Supplementary Table S3 to identify the cut-off values for the five continuous variables: GCS, 14; Albumin, 37.2 g/L; Age, 54 years; AISI, 1,386.37; D-dimer, 2.48 μg/ml.

**Table 2:** Multivariable logistic regression model for predicting LEDVT in aSAH patients.

**Predictor**	**β**	**SE**	***p***	**Odds ratio**
(Intercept)	0.49	1.83	0.79	1.64 (0.04–57.48)
D–dimer	0.25	0.05	< 0.001	1.28 (1.17–1.42)
AISI	0.02	0.01	0.017	1.02 (1.01–1.04)
GCS	−0.2	0.05	< 0.001	0.82 (0.74–0.91)
Age	0.05	0.02	0.002	1.05 (1.02–1.09)
Albumin	−0.12	0.04	0.001	0.89 (0.83–0.96)
MCA	1.15	0.42	0.006	3.16 (1.38–7.18)

GCS, Glasgow coma scale; AISI, aggregate index of systemic inflammation; MCA, middle cerebral artery aneurysm.

### 3.3 Machine learning model performance

**Figure 3:**
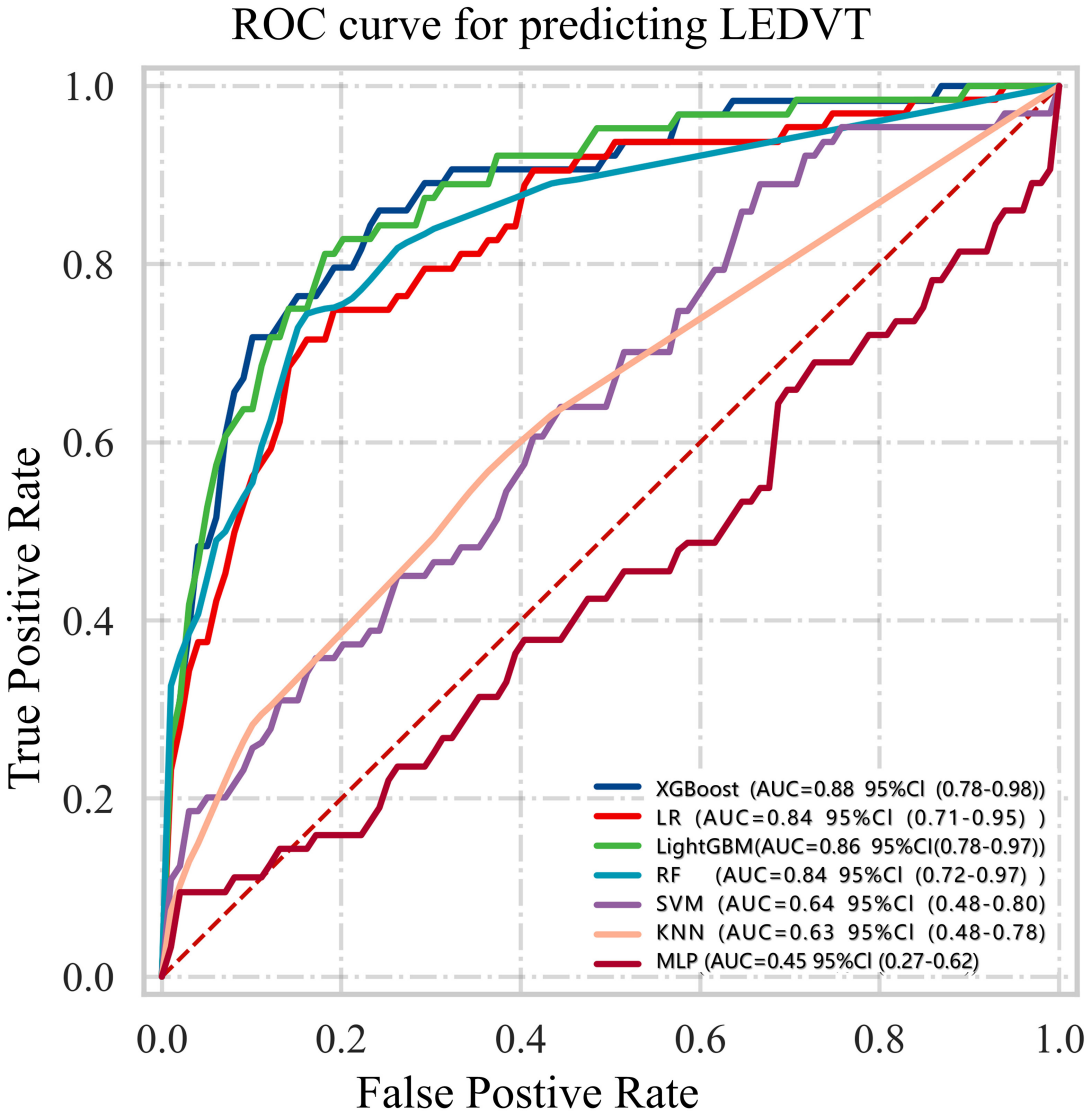
ROC curves for prediction of lower extremity deep vein thrombosis (LEDVT) in the test data set. Greater AUC shows higher discriminative ability of the model.

**Figure 4:**
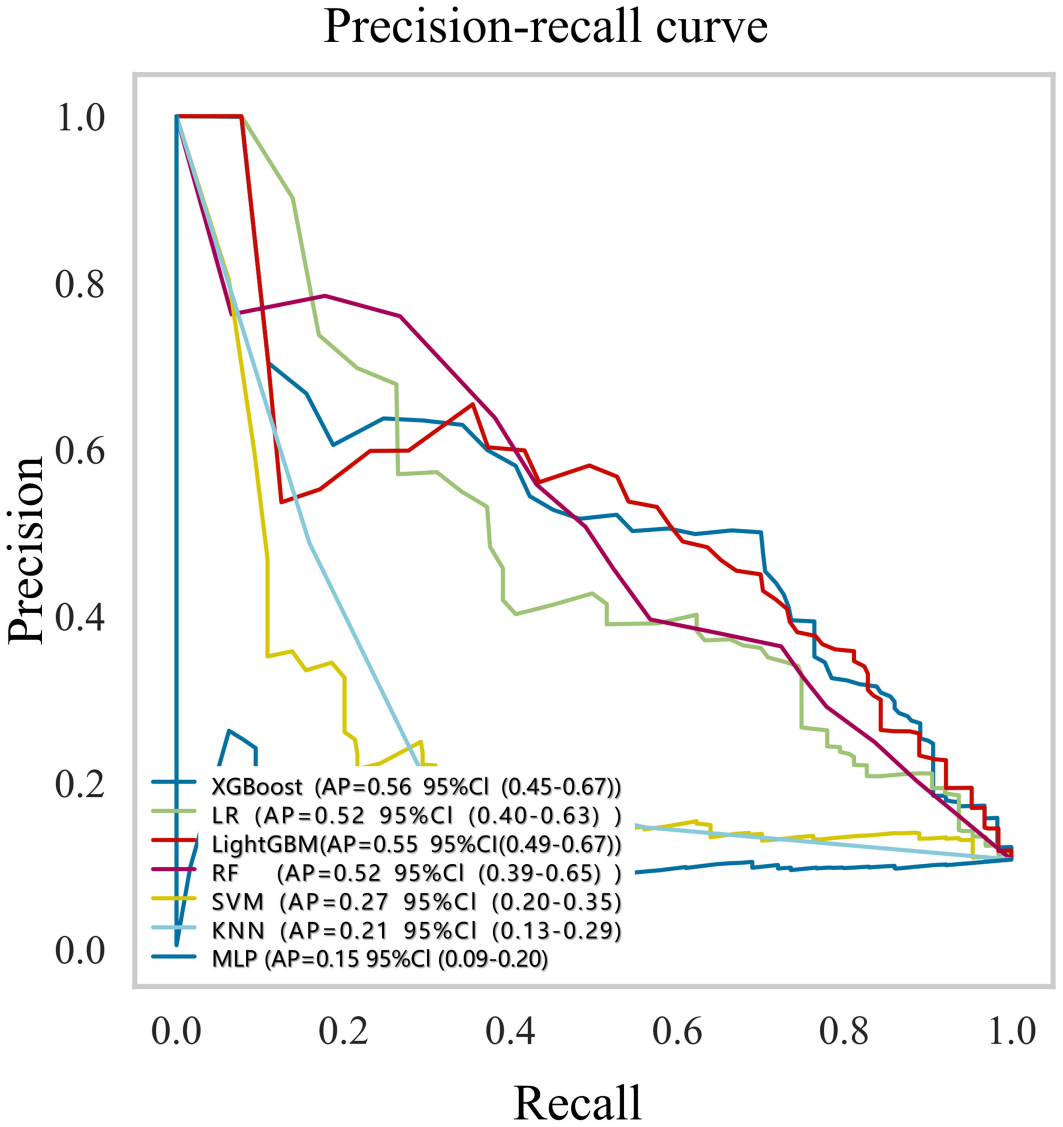
Precision-recall curve for prediction of lower extremity deep vein thrombosis (LEDVT). CI, confidence interval; LR, logistic regression; XGBoost, extreme gradient boosting; LightGBM, light gradient boosting machine; RF, random forest; MLP multilayer perceptron; SVM support vector machine, KNN, K-nearest neighbor.

Based on the six features selected through LASSO regression and multivariable logistic regression screening, we built seven machine learning models: XGBoost, LR, LightGBM, RF, SVM, KNN, and MLP. Supplementary Table S1 and Figure 3 showed the best hyperparameter combination for each model and their AUCs in predicting LEDVT. The AUC values for XGBoost (0.88), LightGBM (0.86), logistic regression (0.84), and random forest (0.84) were notably higher than those for SVM (0.64), KNN (0.63), and MLP (0.45). Among them, XGBoost exhibited the best performance for the prediction of LEDVT risk. As the primary metric, the AUC for XGBoost was 0.88 (95% confidence interval: 0.78–0.98). XGBoost also exhibited the best performance based on the average precision of the precision-recall curve (AP = 0.65, 95% confidence interval: 0.45–0.67) and F1 score (0.46, 95% confidence interval: 0.36–0.56). The Hosmer–Lemeshow goodness of fit test (Supplementary Figure S1) shows a good fit between the XGBoost predicted probabilities and the actual probabilities. Decision curve analysis (Supplementary Figure S2) showed that the XGBoost model provided a greater net clinical benefit than the treat-all or treat-none strategies within the threshold probability ranges of 6%−81% in the training set and 4%−86% in the validation set. Detailed performance metrics, including precision-recall curves and average precision values for all models, are presented in Figure 4 and Table 3. While the AUC-ROC values were excellent, the Area under the precision-recall curve (AUC-PR) and F1 scores were modest (Table 3), which is a common challenge in imbalanced datasets where the positive class is the minority. To further investigate the impact of class imbalance, we performed a sensitivity analysis using resampling techniques. When the XGBoost model was re-trained using SMOTE, the F1 score improved to 0.52 and the AUC-PR increased to 0.70 on the internal test set, albeit with a slight decrease in specificity (from 96% to 92%). Considering that the primary goal of our model is to serve as a high-specificity screening tool to reliably rule out low-risk patients without excessive false alarms, the original model without SMOTE was retained for its superior overall discriminative power (AUC-ROC) and exceptional specificity. The corresponding confusion matrix and performance metrics are provided in Supplementary Table 4.

**Table 3:** Performance of the six ML models in the testing set.

**ML models**	**AUC (95%CI)**	**Accuracy (95% CI)**	**Sensitivity (95% CI)**	**Specificity (95% CI)**	**F1-score**
XGBoost	0.88 (0.78–0.98)	0.9 (0.88–0.91)	0.41 (0.31–0.51)	0.96 (0.95–0.97)	0.46 (0.36–0.56)
LR	0.84 (0.71–0.95)	0.77 (0.71–0.83)	0.76 (0.66–0.86)	0.77 (0.71–0.83)	0.43 (0.32–0.54)
LightGBM	0.86 (0.78–0.97)	0.91 (0.89–0.92)	0.36 (0.20–0.52)	0.97 (0.95–0.99)	0.43 (0.27–0.59)
RF	0.84 (0.72–0.97)	0.9 (0.88–0.92)	0.41 (0.25–0.56)	0.96 (0.94–0.98)	0.45 (0.34–0.56)
SVM	0.64 (0.48–0.80)	0.71 (0.66–0.77)	0.42 (0.30–0.54)	0.75 (0.68–0.82)	0.24 (0.19–0.30)
KNN	0.63 (0.48–0.78)	0.63 (0.61–0.65)	0.57 (0.34–0.80)	0.64 (0.59–0.68)	0.24 (0.16–0.32)
MLP	0.45 (0.27–0.62)	0.87 (0.85–0.89)	0.09 (0.01–0.18)	0.97 (0.96–0.98)	–

LR, logistic regression; XGBoost, extreme gradient boosting; RF, random forest; MLP multilayer perceptron; SVM support vector machine, KNN, K-nearest neighbor.

### 3.4 Application of the model

**Figure 5:**
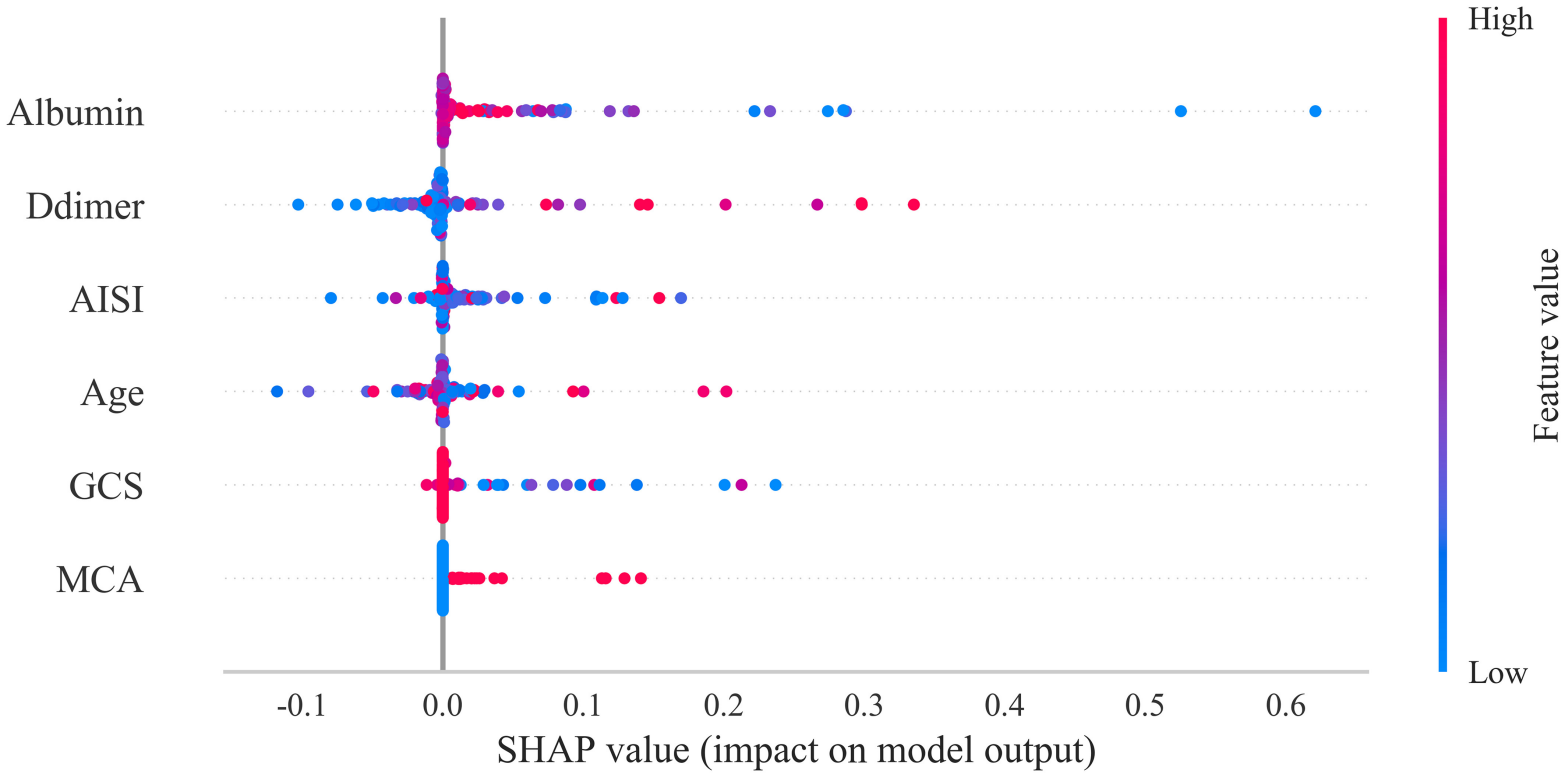
SHAP analysis of the proposed model on the testing set. The SHAP value reflected the impact of features in each sample and performed their positive or negative effects. This figure described data from the testing set, with each point representing one patient. The color represents the value of the variable; red represents the larger value; blue represents the smaller value. The horizontal coordinates represent a positive or negative correlation with DVT risk, with a positive value indicating a risk of DVT and a negative value indicating no risk for DVT. The absolute value of the horizontal coordinate indicates the degree of influence; the greater the absolute value of the horizontal coordinate, the greater the degree of influence. SHAP, shapley additive explanations.

**Figure 6:**
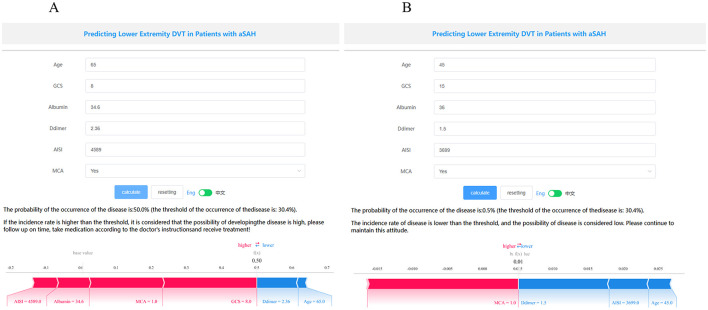
Examples of website usage. Entering the input value determined the transfusion requirements and displayed how each value contributed to the prediction. **(A)** Example 1 has a high probability of LEDVT, the probability of aSAH is 50.0%, and **(B)** Example 2 has a low probability of aSAH, the probability of LEDVT is 0.5%.

The SHAP package conducted a comprehensive analysis of the training set, showing the impact of each variable on predicting LEDVT (Figure 5). The patient characteristics of a patient was input into the model: with age 65 years, the GCS score at admission was 8, albumin level 34.6 g/L, D-dimer level 2.36 ug/ml, AISI 4589, with aneurysm located in the MCA. The model predicted that this patient had a 50.0% risk of developing LEDVT, indicating a high probability and suggesting that preventive interventions should be prioritized (Figure 6A). The another patient was input into the model: with age 55 years, the GCS score at admission was 13, albumin level 36.9 g/L, D-dimer lever 2.68 ug/ml, AISI 3560, without aneurysm located in the MCA. The model predicted a 3.6% risk of LEDVT for this patient, indicating a low probability that does not warrant aggressive preventive measures (Figure 6B). Notably, the threshold of the occurrence of the disease is: 30.4%.

Furthermore, an XGBoost mode website was established to predict LEDVT: https://www.xsmartanalysis.com/model/list/predict/model/html?mid=29059&symbol=8NQ1760Ar952709wO8dX.

### 3.5 External data validation

**Figure 7:**
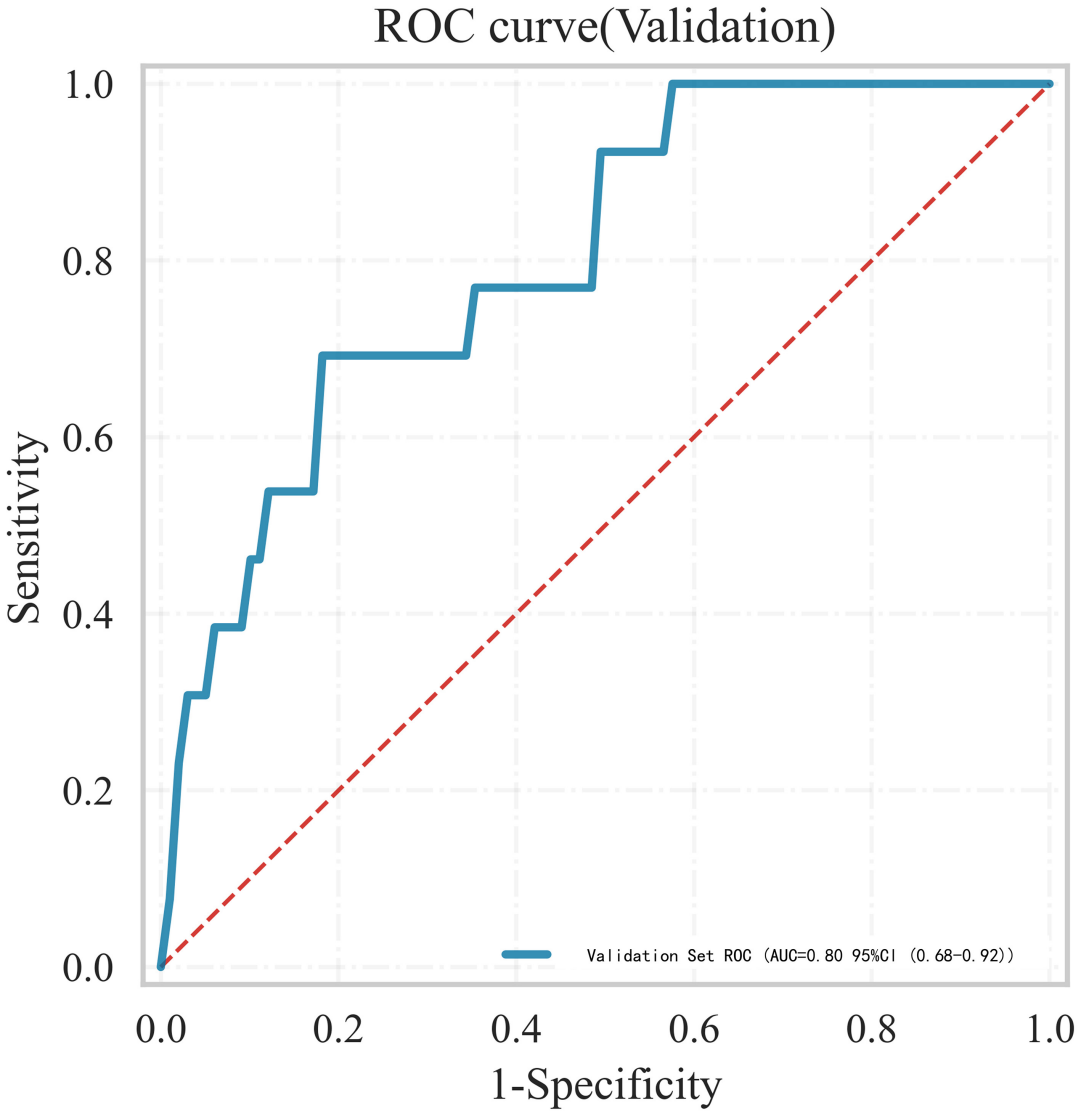
Receiver operating characteristic (ROC) curves for LEDVT in aneurysmal subarachnoid hemorrhage patients in the external validation set.

To further confirm the applicability of our model, we conducted external validation using data from 142 aSAH patients at another hospital. Figure 7 showed that the AUC of the model in the external data is 0.80 (95% confidence interval: 0.68–0.92), indicating that the model can still maintain good performance in the external data.

## 4 Discussion

The incidence of LEDVT in aSAH patients is about 10% to 25% (8, 10, 11). This clinically significant complication increases treatment costs, prolongs hospital stays, and elevates the risk of mortality. As a result, neurosurgeons are increasingly attentive to the prevention and management of LEDVT in clinical practice. In response, this study used seven ML algorithms with clinical and laboratory data to predict the risk of LEDVT for aSAH patients. Ultimately, the XGBoost model was selected as our optimal choice and subsequently validated using aSAH patient data from external hospitals, demonstrating excellent predictive performance for LEDVT.

This study fills an important gap by applying ML algorithms to integrate clinical and laboratory data for LEDVT prediction in aSAH patients. Prior to this study, most research teams relied on traditional statistical models for LEDVT risk prediction. For example, our team previously developed a conventional logistic regression (LR) model to predict LEDVT in aSAH patients. This model was constructed by integrating multiple predictors into a single regression equation and visualizing the results through a nomogram (35). Later, Xu et al. also developed a similar nomogram prediction model to predict LEDVT in aSAH patients, achieving good results (8). Recent evidence, however, suggests that machine learning plays a crucial role in managing postoperative complications in aSAH patients, outperforming conventional prediction models in terms of predictive accuracy. For example, Li et al. applied ML models to predict postoperative pneumonia in aSAH patients and demonstrated that these models outperformed nomograms (AUC: 0.89 vs. 0.85) (15). Similarly, Ping Hu et al. used ML models to predict delayed cerebral ischemia in aSAH patients and reported that both random forest (RF) and artificial neural network (ANN) models (AUC = 0.86) outperformed conventional LR models (AUC = 0.82) (27). All these indicate the significance of machine learning in the perioperative disease management of neurosurgery. Therefore, We used ML to develop models for the prediction of LEDVT for aSAH patients. Model training using data from 593 patients was followed by the 5-fold cross-internal validation. Six algorithms (LR, XGBoost, RF, MLP, SVM, KNN) were used to develop the models, whereas four metrics were used to evaluate their performances. XGBoost model exhibited the best overall performance, with a specificity of 78% and a sensitivity of 94% in predicting LEDVT in aSAH patients.

In our previous work, we established a conventional logistic regression (LR)–based nomogram to predict LEDVT risk in aSAH patients undergoing endovascular treatment, which achieved an AUC of 0.85 (35). In the present study, we referenced that previously developed LR model as a comparator to contextualize the performance of our machine learning (XGBoost) model. The XGBoost model demonstrated superior discriminative ability (Supplementary Figure S3A: AUC: 0.88 vs. 0.85). This enhancement in predictive performance is primarily attributed to the superior capability of ML models in processing high-dimensional data compared to traditional LR methods, thereby enabling the effective incorporation of a broader range of predictive features (36, 37). These findings further support the role of ML in providing individualized risk assessments and serving as a valuable decision-support tool in the clinical management of aSAH. To translate this prediction model into clinical practice, we propose a stratified intervention strategy guided by the estimated risk. For patients with a high predicted risk (≥30%), the model could support the early initiation of pharmacological prophylaxis in the absence of contraindications, while also prompting a careful assessment of the individual's bleeding risk. For those at intermediate risk, intensified mechanical prophylaxis and close monitoring are recommended. This tool, accessible via a web-based calculator, can be integrated into the admission workflow to facilitate timely, data-driven decisions that complement existing clinical protocols for aSAH management.

Additionally, two case examples were presented to demonstrate the predictive output of the XGBoost model and to highlight the relative importance of each clinical variable. Given the high global incidence of LEDVT, the implementation of such predictive tools could assist clinicians in making timely decisions, including the use of prophylactic anticoagulation, early tracheotomy, and infection control strategies.

Furthermore, we noted that prior studies primarily relied on internal validation to assess model performance, which may overestimate generalizability. While internal validation is convenient, it often produces overly optimistic results and exaggerates model performance. Hence, external validation is essential before implementing a predictive model in clinical practice (38). However, current research reveals that most predictive models remain at the development stage, with insufficient emphasis on external validation (39–41). To address this limitation, we conducted external validation using data from another hospital. Although the AUC decreased from 0.88 to 0.80, the model retained acceptable predictive accuracy, supporting its generalizability across institutions. Future efforts should focus on incorporating multi-center data to enhance external validation and improve the model's applicability across diverse patient populations. To promote clinical use and facilitate further validation, we developed an online prediction platform. This tool allows clinicians to estimate LEDVT risk in real-time and contributes external data for ongoing model refinement.

Beyond constructing high-performance predictive models, selecting the right variables is also a critical factor in ensuring the model's effectiveness in practical applications. Clinicians often prefer the simplicity and interpretability of the binning method when applying models in practice (38). However, ML models may become difficult to implement and less practical when they involve too many input variables. Moreover, an excessive number of variables also leads to a decline in the interpretability of the model (42). Therefore, while developing the ML prediction model, we also prioritized the clinical relevance of each selected variable. Many previous studies have conducted extensive research on these predictive variables. Several studies have highlighted the predictive value of these variables. For instance, Wang et al. reported that patients with serum albumin < 35 g/L were more likely to develop preoperative DVT (43). Qin et al. found increased systemic immune-inflammatory index (SII) was correlated with the formation of aSAH-associated DVT after endovascular treatment (11). To avoid missing any valuable predictive variables, we included a total of 37 variables. Comparative analysis revealed that MCA involvement, lower GCS scores, reduced albumin levels, older age, elevated D-dimer levels, and increased AISI were significantly associated with thrombotic events in aSAH patients. Among them, with MCA, lower GCS and albumin level, higher age and D-dimer level have been extensively discussed in many previous articles, and we will not elaborate on them here. Notably, our study introduces a novel composite inflammatory index—the Aggregate Index of Systemic Inflammation (AISI)—which demonstrates a strong correlation with LEDVT. AISI, also termed the pan-immune-inflammation value (PIV), quantifies systemic inflammation by integrating four key complete blood count (CBC) parameters: neutrophils (NEU), platelets (PLT), monocytes (MONO), and lymphocytes (LYM) (44–46). Aneurysmal rupture triggers an intense neuro-inflammatory cascade and a subsequent systemic inflammatory response syndrome (SIRS), characterized by the activation of neutrophils and monocytes, which release pro-coagulant factors and promote platelet adhesion (47, 48). Concurrently, relative lymphopenia indicates immune dysregulation. AISI, by integrating neutrophils, monocytes, platelets, and lymphocytes into a single metric (AISI = neutrophils × monocytes × platelets/lymphocytes), effectively captures this multifaceted interplay of cellular inflammation more holistically than any single cell type or ratio (45). This synergistic dysregulation of multiple cell lineages contributes to endothelial injury and hypercoagulability, creating a fertile ground for venous thrombosis.

While the pathophysiology of aneurysmal subarachnoid hemorrhage (aSAH) remains incompletely understood, emerging evidence implicates dysregulation of immune cells—particularly lymphocytes, neutrophils, and monocytes. Previous studies have reported altered lymphocyte and neutrophil levels in aSAH (11, 49), as well as associations between elevated monocyte counts and adverse outcomes (50). Given these findings, composite indices like AISI, which concurrently evaluate multiple immune components (lymphocytes, neutrophils, platelets, and monocytes), may surpass conventional single-marker indices in clinical utility, as they better capture the complexity of disease-specific inflammatory states.

In most studies, SHAP analyses rely primarily on ranking feature importance, without further exploration of their clinical relevance or translational implications (51). In this study, we first employed LASSO regression to reduce multicollinearity and narrow down the number of candidate variables. We also employed multivariate logistic regression to ensure that each indicator had independent clinical predictive value. Supplementary Table S3 provides cut-off values for each variable to help clinicians better stratify patients by LEDVT risk. Such as the D-dimer level for predicting the LEDVT was 2.48μg/ml, so we need to be more vigilant in preventing DVT for patients with a D-dimer level higher than 2.48. In practice, this value could serve as a red flag, prompting clinicians to intensify monitoring (e.g., more frequent lower extremity Doppler ultrasounds) or to re-evaluate the benefit-risk ratio of initiating or escalating prophylactic anticoagulation, especially when other risk factors are present. However, AISI alone yielded an AUC of only 0.60, indicating limited standalone predictive value. Notably, removing AISI from the model—even due to its modest individual AUC—would reduce overall model performance from 0.88 to 0.74 (Supplementary Figure S3A). To further validate the independent value of AISI beyond its individual components, we conducted a sensitivity analysis. We constructed an alternative XGBoost model wherein AISI was replaced by its constituent parts (neutrophil count, monocyte count, platelet count, lymphocyte count) and other common inflammatory markers (NLR, SII, SIRI, CRP). The model retaining AISI achieved a superior performance (AUC: 0.88) compared to the alternative model without AISI (AUC: 0.82). This comparative analysis confirms that AISI provides unique predictive information that is not fully captured by its components or related indices alone, underscoring its value as an integrative marker of systemic inflammation in our prediction model. This underscores the importance of accounting for interactions between variables—one of the key advantages offered by machine learning in clinical prediction modeling. This approach not only ensures the simplicity of the model's operation (requiring only the input of six easily obtainable clinical variables), but also takes into account the accuracy and effectiveness of the model's predictions.

In brief, these findings indicate that the model possesses not only internal applicability within our institution but also extensive generalizability across other healthcare facilities.

This study has several limitations that should be considered. First, its retrospective nature inherently introduces the potential for selection bias and unmeasured confounding, despite our efforts to adjust for known variables. Second, the data were sourced exclusively from centers in a single country (China), and the genetic, lifestyle, and clinical management homogeneity of this population may limit the model's generalizability to other ethnic and healthcare settings. Third, while external validation is a key strength, the cohort from the external center was relatively small (*n* = 142), which may affect the stability of the performance estimates. Fourth, our model predicts the risk of LEDVT within a 30-day postoperative window. While this period captures the peak incidence of this complication, the risk of venous thromboembolism may persist beyond this timeframe, especially in patients with prolonged immobility or other ongoing risk factors. Our model's performance in predicting long-term risk remains unknown. Additionally, our study is limited by the class imbalance inherent in the clinical occurrence of LEDVT, with only 10.8% of patients in the primary cohort developing the outcome. This imbalance contributed to the disparity between the strong AUC-ROC and the more modest AUC-PR and F1 scores, as the latter metrics are more sensitive to the performance on the minority class. Although we used stratified cross-validation and conducted external validation to mitigate overfitting, and our sensitivity analysis with resampling showed some improvement, the model's sensitivity remains moderate. This indicates a trade-off whereby the model is excellent at identifying low-risk patients (high specificity) but may miss a portion of true high-risk cases. In clinical practice, this suggests that the model is best used as a high-specificity screening tool to rule out low-risk patients, while traditional risk factors and clinical vigilance should still be applied universally. Future work should involve prospective collection of larger, multi-center datasets with more balanced classes or the application of advanced cost-sensitive learning algorithms to improve the detection of minority-class patients without compromising overall performance

## 5 Conclusion

We developed and externally validated a machine learning model, with XGBoost showing optimal performance in predicting LEDVT risk after aSAH. By integrating key clinical and inflammatory variables, the model demonstrated strong predictive power and generalizability. The inclusion of AISI underscores the importance of systemic inflammation in thrombosis risk. A user-friendly web tool was also established to support clinical decision-making. This model may aid early identification and personalized prevention of LEDVT in aSAH patients.

## Data Availability

The raw data supporting the conclusions of this article will be made available by the authors, without undue reservation.
