# Supplementary material for: Predicting lower extremity deep venous thrombosis in patients with aneurysmal subarachnoid hemorrhage: a machine learning study
Source: Front Neurol. 2025 Nov 24;16:1659212. doi: 10.3389/fneur.2025.1659212 (PMC12682691; doi:10.3389/fneur.2025.1659212)
Supplement: Supplementary file 1 [file Table_1.doc]

**Supplementary information**

**Table S1. Hyperparamter combinations for each machine learning models and their corresponding test set AUC**

|  | **Hyperparameters Set** | **Optimal Hyperparameters** | AUC |
| --- | --- | --- | --- |
| LR | C: 10.0 max iter: 100 penalty: l2 tol: 0.0001 | L1 Regularization term: 10.0 | 0.915 |
| SVM | Kernel:{Linear, Polynomial, Radial Basis, Sigmoid} Regularization term: {0,1,2,3,4,5} | Kernel: Radial Basis Regularization term: 1 | 0.896 |
| RF | Criterion: gini max_depth: None min impurity decrease: 0.0 n estimators: 100 | Number of estimators: 100 Maximum depth: None | 0.994 |
| KNN | Number of neighbors: 6 weights: distance | Number of estimators: 500 Learning rate: 0.05 | 0.995 |
| MLP | activation: logistic hidden layer sizes: (30, 30) max iter: 10 | L2 Regularization term: 0.1 | 0.671 |
| XGB | Number of estimators: {50,100,200,500} Learning rate: {0.1,0.05,0.01} Maximum depth: {3,4,5,6} | objective: binary:logistic learning rate: 0.3 max depth: 6 min child weight: 2 L2 Regularization term: 1 | 0.970 |
| Light GBM | Number of estimators: {50,100,200,500} Learning rate: {0.1,0.05,0.01} Maximum depth: {3,4,5,6} | objective: binary:gbdt learning rate: 0.1 max depth: -1 n_estimators: 100 num_leaves: 31 | 0.972 |

Note: LR, logistic regression; SVM, support vector machine; RF, random forest; MLP, multilayer perceptron; KNN, K-nearest neighbor;; XGB, extreme gradient boosting. AUC score measured the model performance on testing set.

**Table S2.** TRIPOD checklist

| Section | Item | Checklist Item | Reported (Yes/No) | Location / Notes |
| --- | --- | --- | --- | --- |
| Title/Abstract | 1 | Identify study as developing/validating prediction model | Yes | Title and Abstract |
| Introduction | 2 | Medical context and rationale | Yes | Introduction, Caprini limitations |
| Introduction | 3 | Specify objectives (develop/validate) | Yes | End of Introduction |
| Methods: Source of Data | 4a | Study design and data source | Yes | Methods, Study Population |
| Methods: Source of Data | 4b | Key study dates | Yes | Methods, January 2020 – December 2022 |
| Participants | 5a | Inclusion/exclusion criteria | Yes | Methods section |
| Participants | 5b | Study setting | Yes | Neurosurgery departments |
| Participants | 5c | Dev/val datasets | Yes | Internal: 593, External: 142 |
| Outcome | 6a | Outcome definition | Yes | LEDVT confirmed by DUS within 30 days |
| Predictors | 7a | Definition of predictors | Yes | Age, GCS, Albumin, D-dimer, AISI, MCA |
| Sample Size | 8 | Sample size determination | Yes | Explicitly |
| Missing Data | 9 | Handling of missing data | Yes | Delete |
| Statistical Analysis | 10a | Handling of predictors | Yes | LASSO, logistic regression |
| Statistical Analysis | 10b | Model-building procedures, validation | Yes | 7 ML models, 5x5 CV |
| Statistical Analysis | 10c | How predictors entered | Yes | ROC cut-offs listed |
| Statistical Analysis | 10d | Performance measures | Yes | AUC, F1, AP, Kappa |
| Statistical Analysis | 10e | External validation method | Yes | Separate 142-patient cohort |
| Results | 13a | Participant flow and attrition | Yes | Methods and Figure |
| Results | 13b | Participant characteristics | Yes | Table 1 |
| Model Development | 14a | Number of events and predictors | Yes | 64 events, 6 predictors |
| Model Development | 14b | Unadjusted associations | Yes | Table 2 |
| Model Specification | 15a | Present full model (equation or calculator) | Yes | Web-based calculator provided |
| Model Specification | 15b | Explain model use | Yes | Case examples provided |
| Model Performance | 16 | Report performance measures | Yes | AUC, AP, F1 with 95% CI |
| Discussion | 18 | Study limitations | Yes | Single-center, AISI alone not sufficient |
| Discussion | 19b | Overall interpretation | Yes | ML adds value in risk stratification |
| Discussion | 20 | Clinical use and future research | Yes | Online tool, multicenter expansion |
| Other Information | 21 | Supplementary info or data availability | Yes | Web tool provided |
| Other Information | 22 | Funding and role of funder | Yes | Jinhua Science and Tech Bureau |

Table S3. AUC, Sensitivity, Specificity, and Cut-off values for each Characteristics in the model

| Characteristics | AUC(95%CI) | Sensitivity (95% CI) | Specificity (95% CI) | Cut-off value (95% CI) |
| --- | --- | --- | --- | --- |
| GCS | 0.74(0.69-0.78) | 0.82(0.78-0.84) | 0.64(0.56-0.72) | 14(14-15) |
| Albumin (g/L) | 0.65(0.57-0.72) | 0.73(0.65-0.94) | 0.58(0.33-0.69) | 37.20(33.75-38.00) |
| Age | 0.65(0.59-0.70) | 0.78(0.43-0.89) | 0.47(0.41-0.82) | 54(52-68) |
| AISI | 0.60(0.53-0.67) | 0.45(0.22-0.85) | 0.73(0.30-0.95) | 1386.37(477.65-3730.30) |
| D-dimer(µg/mL) | 0.82(0.76-0.86) | 0.75(0.58-0.81) | 0.77(0.75-0.91) | 2.48(2.48-3.68) |

Table S4: Performance comparison of the XGBoost model after using resampling technology.

| TechniqueAUC-ROC (95% CI)AUC-PRF1-ScoreSensitivitySpecificityOriginal (No Resampling)0.88 (0.78-0.98)0.650.460.410.96SMOTE (After resampling)0.85 (0.74-0.96)0.700.520.580.92 | |  | |  | |  | |  | |  |
| --- | --- | --- | --- | --- | --- | --- | --- | --- | --- | --- |
|  |  | |  | |  | |  | |  | |
|  |  | |  | |  | |  | |  | |

Table S4. Performance comparison of the XGBoost model when trained with different resampling techniques on the internal training cohort and evaluated on the internal testing set. SMOTE: Synthetic Minority Over-sampling Technique.
